# Supplementary material for: AI-Augmented Quantitative MRI Predicts Spontaneous Intracranial Hypotension
Source: Diagnostics (Basel). 2025 Sep 15;15(18):2339. doi: 10.3390/diagnostics15182339 (PMC12468366; doi:10.3390/diagnostics15182339)
Supplement: Supplementary file 1 [file diagnostics-15-02339-s001.zip › diagnostics-3754781-supplementary.pdf]

Supplementary Table S1. CSF flow biomarkers across aqueduct vs cervical studies. CSF flow biomarkers reported in SIH across intracranial aqueduct versus upper-cervical (C2–C3) sites, plus HV normative ranges. Studies are grouped by measurement site to highlight divergent directions (↓ vs ↑) reported in the literature.

**Study (year)**	**Site**	**N (SIH / HV)**	**Key metric(s)**	**Direction in SIH vs HV**	**Pre/Post change**
Yang et al., 2014	Aqueduct	20 / 31	Average CSF flow (μL/beat), peak velocities; ↑ after tx	↓ (SIH < HV)	↑ SIH CSF flow post-treatment
Tung et al., 2014	Aqueduct	32/17	PC-MR flow parameters for diagnosis & follow-up	↓ (SIH < HV)	Cross-sectional
Tsai et al., 2018	C2/C3	28/20	CSF flow (ml/s)	↓ (SIH < HV)	↑ SIH CSF flow post-treatment
Wolf et al., 2023	C2/C3 & C5/C6	20 / 40	CSF velocity	↑ (SIH > HV)	Cross-sectional
Wolf et al., 2024	C2/C3	117 / 70	CSF velocities	↑ (esp. lateral leaks/CSF-venous fistula)	Cross-sectional
Beltrán et al., 2023	C2/C3 & C5/C6)	0 / 70 (HV)	C2/C3: CSF velocity range 4.5±0.9 cm/s; CSF flow-rate range 8.6±2.4 mL/s; CSF stroke volume 2.1±0.7 mL. C5/C6 vs C2/C3: velocity +1.64 cm/s; stroke volume −0.4 mL.	N/A (HV normative)	—

Abbreviations: HV, healthy volunteers. N/A: non applicable.

Supplementary Table S2. Comparison of AI and Conventional Methods for CSF Tissue Segmentation and PC-MRI CSF Flow Analysis (Brain and Spine).

Column guide: Domain (brain/spine/flow), Task/Modality, Method Type, Representative Architecture/Technique, Key Performance (as reported), Vs. Manual/Conventional, Reference (Ref key).

Domain	Task / Modality	Method Type	Representative Architecture / Technique	Key Performance (as reported)	Vs. Manual / Conventional	Reference (Ref key)
Brain	CSF tissue segmentation / MRI	Deep learning (CNN)	Encoder–decoder; transfer learning (e.g., from existing algorithms/VGG family)	Clinical datasets often report Dice > 0.8 (overall trend)	End-to-end semantic segmentation; improved efficiency & reproducibility	Grimm 2020 (Acta Neurochirurgica)
Brain	CSF tissue segmentation / MRI	Deep learning (CNN; U-Net variants)	U-Net variants; end-to-end quantification	Dice > 0.8 (reported)	Enables automated quantification of edema & CSF distribution	Cui 2024 (Heliyon)
Brain	Intracranial compartments & CSF volumetry / CT	Deep learning (segmentation networks)	Automated volumetric pipeline	Demonstrated clinical feasibility; mass-effect quantification	Supports near real-time, objective assessment	Puzio 2024 (Frontiers in Neuroscience)
Brain	Brain/CSF segmentation / MRI (3D)	Multiple-classifier system (ensemble MLPs)	Majority voting; 3D features	Robust under noise/intensity inhomogeneity	More robust than single classifiers	Amiri 2017 (MBEC)
Brain	Brain parenchyma & CSF / multispectral MRI	Classical (EM clustering, thresholding, connectivity)	Unsupervised + anatomical priors/post-processing	Computationally efficient; accuracy limited in complex pathology	Often used as baseline or auxiliary methods	Lundervold 1995 (IEEE TMI)

Brain	CSF/GM/WM segmentation / unenhanced CT	Classical (rule/threshold/statistical)	Traditional image-processing pipeline	Demonstrated feasibility of automatic segmentation	Reduces manual workload	Gupta 2010 (Academic Radiology)
Spine	Vertebral/spinal structures (incl. CSF boundary) / MRI	Deep learning (CNN)	Semantic segmentation; multi-center, multi-parametric	Clinical applicability across centers	Preserves boundary details; scalable	Sáenz-Gamboa 2023 (AI in Medicine)
Spine	Spinal cord/CSF region segmentation / MRI	Deep learning (multi-path dense network)	Multi-path dense network	Good segmentation with boundary preservation	Performs well in small-scale/low-contrast settings	Liang 2021 (PLOS ONE)
Spine	Spinal cord segmentation (topology-consistent) / MRI	Atlas-based + topology constraints	Deformable atlas + topology-preserving mechanisms	Improved anatomical plausibility	Better repeatability & consistency	Chen 2013 (NeuroImage)
Brain	Brain/CSF segmentation / MRI T1	Classical (enhanced fuzzy C-means)	Enhanced spatial FCM	Improved spatial consistency vs. standard FCM	More robust than standard FCM	Jafrasteh 2024 (Neuroinformatics)
Brain (flow)	PC-MRI aqueduct auto-segmentation + flow quantification	Deep learning (CNN: U-Net / MultiResUNet)	Automatic ROI; end-to-end segmentation + flow metrics	Dice ≥ 0.93; lower segmentation-failure rate; superior flow-parameter agreement	Reduces inter-/intra-operator variability; improves efficiency & reproducibility	Tsou 2021 (J Clin Neurosci)
Brain (flow)	PC-MRI flow quantification (semi-automated)	Semi-automated (seed-based/contouring)	Semi-automated ROI tracking	ICC significantly higher than fixed-radius approach	Mitigates subjective bias; improves consistency	Flórez 2006 (MAGMA)
Brain (flow)	Methodological sensitivity in 2D PC-MRI	Non-AI; methods evaluation	ROI area/placement	ROI differences significantly	Underscores need for automation	Zhang 2023 (Acta Radiologica)

			affects stroke volume	impact stroke volume		
Brain (flow)	Normal aqueduct & perivascular CSF flow / 2D PC-MRI	Non-AI quantification study	2D PC-MRI pipeline	Established normal ranges & feasibility	Serves as clinical baseline/benchmark	Le 2025 (Frontiers in Neuroscience)
Spine (flow)	PC-MRI spinal CSF flow & cord motion	Non-AI (current baseline)	ROI-based dynamic flow and displacement measurements	Reference ranges in healthy participants (n=70)	Baseline for future AI development	Beltrán 2024 (NMR in Biomedicine)

Abbreviations: CNN, convolutional neural network; DL, deep learning; ROI, region of interest; ICC, intraclass correlation coefficient; FCM, fuzzy C-means; GM/WM, gray/white matter; PC-MRI, phase-contrast MRI.

Supplementary Table S3. Vertebral levels of the first epidural blood patch by outcome group. The distribution of thoracic versus lumbar levels did not differ between groups (Fisher's exact p = 0.64).

**Group**	**Cervical (n, %)**	**Thoracic (n, %)**	**Lumbar (n, %)**	**Total**
First-EBP success	0 (0.0%)	9 (69.2%)	4 (30.8%)	13
First-EBP failure	0 (0.0%)	10 (83.3%)	2 (16.7%)	12
Total	0 (0.0%)	19 (76.0%)	6 (24.0%)	25
